# Impact of Metagenomic Next-Generation Sequencing on Antibiotic Management in Pediatric Patients

**DOI:** 10.3390/medicina62030482

**Published:** 2026-03-04

**Authors:** Ariel Gonzalez, Jill Argotsinger, Ronda J. Oram, Jessica L. Miller

**Affiliations:** 1 Department of Pharmacy, Advocate Lutheran General Hospital, Park Ridge, IL 60068, USA; ariel.gonzalez@aah.org (A.G.);; 2 Department of Pediatrics, Advocate Children’s Hospital, Park Ridge, IL 60068, USA

**Keywords:** pediatrics, diagnostics, stewardship, metagenomic next-generation sequencing, immunocompromised

## Abstract

*Background and Objectives*: Metagenomic next-generation sequencing (mNGS) is an emerging diagnostic tool used to guide the management of infectious diseases. However, clinical criteria in which there is a clear benefit have not been identified, and more real-world clinical experience is needed to identify patient populations in which mNGS testing may have the most benefit. The aim of this article is to evaluate the utilization of mNGS to determine the impact on clinical practice for pediatric patients. *Materials and Methods*: This retrospective analysis included pediatric patients that had a mNGS test performed between January 2020 and September 2024. The primary outcome was the clinical impact of the mNGS test on patient management defined as either a positive impact or no impact. Secondary outcomes included test turnaround time, agreement or discordance between conventional testing and mNGS, and hospital length of stay. *Results*: Forty-six mNGS tests in 42 patients were evaluated. Of 60 organisms identified from the 46 tests, 27 organisms (45%) were considered clinically significant. mNGS had a positive clinical impact in 18 (39.1%) patients, primarily due to antimicrobial modifications (16, 34.8%) and new diagnoses (6, 13.0%). The majority of patients with a positive clinical impact were immunosuppressed (15/18, 83.3%). *Conclusions*: mNGS demonstrated utility in a subset of pediatric patients, particularly those considered immunosuppressed. Its ability to confirm or exclude infections, particularly fungal infections in this patient population, contributed to its impact. However, its limited benefit in immunocompetent patients underscores the importance of careful patient selection to optimize diagnostic and antimicrobial stewardship.

## 1. Introduction

Infectious diseases remain a leading cause of morbidity and mortality in pediatric patients, particularly in vulnerable populations, such as those who are immunosuppressed [1]. Additionally, pathogen detection remains challenging, as traditional diagnostic methods fail to identify a pathogen in up to 60% of infectious disease cases, especially in settings where cultures cannot be reliably obtained and/or have low yield or the causative organism is difficult to culture [2]. Metagenomic next-generation sequencing (mNGS) is a relatively recent advancement in diagnostic technology that has provided clinicians with a novel way to detect pathogens. Unlike conventional microbiological tests (CMT), which rely on cultures, serologies, or polymerase chain reactions (PCR), mNGS offers a broad analysis of all genetic material in a sample, thus enabling the identification of bacteria, viruses, fungi, and parasites [3]. This makes mNGS especially valuable compared to CMT in certain cases, such as infections that are difficult to diagnose, caused by a fastidious or rare pathogen, or polymicrobial [4,5]. Furthermore, mNGS is a non-invasive blood test and may be advantageous in children where invasive procedures, such as biopsies or bronchoscopies to collect samples for pathogen identification for CMT, are not always desired or feasible [4].

Compared to traditional diagnostic approaches, mNGS may offer a more sensitive method for identifying infectious organisms in specific disease states. Potential benefits of mNGS have been demonstrated in multiple clinical syndromes, including diagnosis of fungal pathogens in immunosuppressed hosts and pathogen identification for infective endocarditis [6,7,8]. Testing has also shown a benefit in children with pneumonia, febrile neutropenia, and fever of unknown origin [9,10,11,12,13].

Despite the advantages of mNGS, there are also challenges and limitations. The test is associated with a significant financial cost that may limit its ability to be used routinely. Additionally, the need to send specimens to a reference lab can lead to delays in test results. Another concern is the potential for over-detection of commensal organisms that may not be pathogenic [5]. Lastly, clinical criteria in which there is a clear benefit have not been identified [14,15].

Data is still emerging on the application of mNGS, specifically in the pediatric patient population. This analysis aims to evaluate the utilization of mNGS testing to determine the clinical impact to optimize its use in pediatric patients.

## 2. Materials and Methods

This analysis was conducted at Advocate Children’s Hospital, consisting of two campuses within community teaching hospitals with affiliated outpatient clinics (Oak Lawn, IL, USA and Park Ridge, IL, USA). Pediatric patients < 18 years old were included if they had a plasma mNGS test collected between January 2020 and September 2024. Patients were identified from an electronic health record (EHR) report, and manual chart review was subsequently performed for each test to gather patient baseline characteristics and collect data points relevant to primary and secondary outcomes.

At our institution, ordering of a plasma mNGS test (Karius Spectrum^®^, Redwood City, CA, USA) can only be done by or in consultation with an infectious disease clinician. This is a blood test based on metagenomic sequencing of plasma microbial cell-free DNA that can identify 1250 pathogens, including fungi, bacteria, parasites, and viruses. Whole blood specimens are collected in a plasma preparation tube and then centrifuged within 6 h of draw at 1100 RCF (g) for 10 min at ambient temperature to separate the plasma from the cells. The preparation is then delivered to an external laboratory (Karius Laboratory, Redwood City, CA, USA) for sequencing and analysis per routine testing protocols. The cell-free DNA are extracted and compared against a proprietary reference genome database. Results, including the microorganisms detected, the DNA molecules per microliter for quantitative concentrations, and the reference interval, are sent back to the provider and uploaded onto the EHR. The analytical specificity and sensitivity of this test are >99% and >95%, respectively [16].

The primary outcome of this retrospective analysis was to determine clinical impact defined as either a positive impact or no impact. A positive clinical impact was determined if the mNGS result led to a new diagnosis based on provider documentation and/or if an antimicrobial was added (antibiotic, antifungal, antiviral) and/or modified (escalated, de-escalated, discontinued, dose adjusted). No impact was determined if the mNGS result confirmed a diagnosis made based on CMT and/or if no changes were made to antimicrobial management. The primary outcome was further stratified by whether patients were immunosuppressed. Patients were considered immunosuppressed if they had an underlying primary immunodeficiency, underwent an organ or stem cell transplant, were on long-term steroids, or had a hematological malignancy with or without active chemotherapy. An infectious disease physician was available to review patients if impact was unclear if needed.

Secondary outcomes included test turnaround time, agreement or discordance between clinically significant CMT and mNGS results, and length of hospital stay. Test results were considered to have positive agreement if both CMT and mNGS identified at least one identical, clinically relevant organism. They were considered to have negative agreement if neither detected a clinically relevant organism. Lastly, testing was considered discordant if there was a mismatch between CMT and mNGS results for clinically relevant organisms. Indication and clinically significant results were determined based on infectious disease provider documentation in the EHR. Results of mNGS tests were deemed clinically significant if the provider documented that the organisms identified were a presumed pathogen as opposed to commensal organisms. Analyses were performed using SAS Enterprise Guide 8.6 (SAS Institute Inc., Cary, NC, USA). Descriptive statistics were performed for baseline characteristics and outcomes. Continuous variables are reported as medians and interquartile ranges. Categorical variables are reported as frequencies and percentages unless otherwise noted. No comparative analyses were performed. Our protocol was reviewed by our Institutional Review Board, and the need for informed consent was waived as the protocol was deemed not human subjects research.

## 3. Results

A total of 46 mNGS tests in 42 patients were assessed during the designated time frame. Four patients had two mNGS tests that were sent upon different hospital admissions for separate infection evaluations. Therefore, these separate infection evaluations were evaluated independently as separate events in our analysis. No patients had more than one mNGS test sent during the same hospital stay. There were equal numbers of males and females (23, 50%) with a median age of 8.5 years (IQR, 2.3–14.8). The majority of patients were immunosuppressed (27, 58.7%), with most undergoing chemotherapy for hematological malignancies (21/27, 77.8%). Similar numbers of patients were in the intensive care unit (ICU) (19, 41.3%) vs. non-ICU (26, 56.5%), with mNGS testing performed outpatient for 1 (2.2%) patient. Most had antimicrobials administered empirically (39, 84.8%), with approximately a third of patients (16, 34.8%) having a definitive diagnosis prior to mNGS results. mNGS was performed most frequently for pulmonary infection (12, 26.1%), febrile neutropenia (11, 23.9%), and fever of unknown origin (9, 15.6%). Additional baseline characteristics can be found in Table 1.

A total of 60 organisms were identified through mNGS, of which 27 (45%) were considered clinically significant per physician documentation. Among these, 22/27 (81.5%) were identified in immunosuppressed patients. Of the organisms identified in this subgroup, 11/22 (50%) were bacteria, 7/22 (31.8%) were fungi, and 4/22 (18.2%) were viruses (Table 2).

For the primary outcome, mNGS testing had a positive clinical impact in 18 (39.1%) patients. Details of the 18 patients with a positive impact can be found in Table 3. A new diagnosis was made in 6 (13.0%) patients, antimicrobials were added in 1 (2.2%) patient, and antimicrobials were modified in 16 (34.8%) patients. Of the antimicrobials modified, the majority were either de-escalations (9, 19.6%) or discontinuations (9, 19.6%). Importantly, most cases with a positive clinical impact were in immunosuppressed patients (15/18, 83.3%), which included 6 (13%) patients with a new diagnosis, 1 (2.2%) patient with antimicrobials added, and 13 (28.3%) patients with antimicrobials modified (Table 4). mNGS was deemed to have no impact for 27 patients (58.7%). When stratified by type of infection, mNGS testing had a positive impact in 7/11 (63.6%) and 5/12 (41.7%) of cases sent for febrile neutropenia and pulmonary infections, respectively (Table 5). Notably, all but one of these cases involved immunosuppressed patients.

The majority of CMT and mNGS test results had a negative agreement (26, 56.5%). Positive agreement was observed in 8 (17.4%) patients and discordant results in 12 (26.1%) patients. Of the 12 discordant results, 4 patients had a fungal pathogen identified on mNGS that was not identified on CMT. Median length of hospital stay was 21 days (IQR, 13–35), and median mNGS test turnaround time was 4.5 days (IQR, 3.0–6.8).

## 4. Discussion

Our findings demonstrate that mNGS testing made a positive impact on clinical management in pediatric patients, particularly in immunosuppressed patients with pulmonary infections or febrile neutropenia. In this analysis, mNGS tests led to a positive clinical impact in 18/46 (39.1%) patients, with 16/46 (34.8%) cases involving antimicrobial modifications and 6/46 (13.0%) cases involving a new diagnosis, which is the same or higher than previously reported studies [3,4,12,14]. Notably, we did not include the administration of unnecessary antimicrobial therapy in our analysis. Future studies should explore the potential for unnecessary treatment of commensal organisms due to difficulty interpreting mNGS results.

The impact of mNGS was most pronounced among immunosuppressed patients in our analysis. Among the 18 patients who had a positive clinical impact from testing, 15 (83.3%) were immunosuppressed, including 6/15 (40%) new infectious diagnoses and 13/15 (86.7%) antimicrobial modifications. The literature is conflicted on the benefit in this patient population. One retrospective study on pediatric immunosuppressed patients found that mNGS testing led to changes in clinical management in 14/104 (13%) cases [14]. Another study concluded that use in immunosuppressed patients was associated with more positive impacts compared to those that were non-immunosuppressed (67/247, 27.1% vs. 139/1226, 11.3%; *p* < 0.001) [4]. In our analysis, the greatest benefit was seen in detecting fungal pathogens. There were seven clinically significant fungal organisms that were identified in five patients. This led to three new Aspergillosis diagnoses and one new Candidiasis diagnosis. Additionally, mNGS results led to antifungal de-escalation or discontinuation in 11/18 (61%) patients who experienced a positive clinical impact. There were five patients who were started on amphotericin B empirically and subsequently transitioned to a narrower spectrum antifungal once mNGS results arrived. A negative test for a fungal pathogen also led to antifungal discontinuation in six patients. This suggests that mNGS testing may be valuable when a fungal infection is considered in an immunosuppressed patient. However, the role of mNGS testing needs further exploration in this setting, as a negative test does not rule out invasive fungal disease. There was limited value in obtaining mNGS testing for non-immunosuppressed patients in our analysis, as there was no impact for 16/19 (84%) patients. Larger studies may be needed to determine if there is a benefit in this patient population for certain indications.

The literature varies on the agreement of mNGS results compared to CMT [2,4,11,14]. We observed discordance between mNGS and CMT results in 12 (26.1%) cases. Of those, mNGS results led to a positive impact in nine patients, with four patients receiving a new fungal diagnosis. Furthermore, we observed a negative agreement in which neither detected a clinically relevant organism in 26/46 (56.5%) cases. Both scenarios highlight the role that mNGS may play in antimicrobial stewardship in addition to avoiding invasive diagnostic procedures when the yield for CMT is low. However, there is also the potential for overuse of mNGS testing when a diagnosis is confirmed by CMT, as 34.8% and 15.2% of patients had a diagnosis and completed treatment before mNGS results in our analysis. Careful consideration should be made in determining when mNGS testing can provide the most benefit compared to when CMT can be utilized.

The median time to mNGS testing was 6 days, with a median turnaround time of 4.5 days, suggesting an opportunity for earlier testing in select patient populations where it may offer the greatest benefit. Additionally, with a median length of stay of 21 days, some clinicians may consider keeping patients admitted while awaiting mNGS results. Balancing the potential benefits of inpatient monitoring with hospital resource utilization remains an important consideration.

This analysis has several limitations. First, its retrospective design, conducted at two pediatric campuses, may limit the generalizability of its findings. The relatively small sample size further restricts the ability to draw definitive conclusions, and larger studies may be needed to describe the full benefits of mNGS testing. Second, our definition of clinically significant organisms relied on physician documentation. This introduces potential bias in the diagnosis of an active infection given mNGS results may be more difficult to interpret compared to CMT due to differences in sensitivity, capability for organism detection independent of viability, and considerations of specimen quality. Additionally, we did not conduct a cost–benefit analysis, which may provide further insight into decision making due to the financial impact of this test. Further studies are needed to better characterize the patient populations most likely to benefit from mNGS and assess its financial impact.

## 5. Conclusions

This retrospective analysis demonstrates that mNGS can have a positive clinical impact in pediatric patients, specifically those who are immunosuppressed when there is a concern for a fungal infection. mNGS represents a potentially valuable diagnostic tool to consider when managing complex infectious diseases in certain clinical scenarios.

## Figures and Tables

**Table 1:** Baseline characteristics.

Patient Characteristics (*n* = 46)	
Age in years	
Median age (IQR)	8.5 (2.25–14.75)
Sex, *n* (%)	
Male	23 (50.0)
Female	23 (50.0)
Race, *n* (%)	
White	34 (73.9)
Black/African American	10 (21.7)
Asian	2 (4.3)
Ethnicity, *n* (%)	
Not of Hispanic/Latino origin	30 (65.2)
Hispanic/Latino origin	16 (34.8)
Immune condition, *n* (%)	
Hematological malignancy on chemotherapy	21 (45.7)
Hematological malignancy not on chemotherapy	1 (2.2)
Immunodeficiency	5 (10.9)
Patient location, *n* (%)	
Non-intensive care unit	26 (56.5)
Intensive care unit	19 (41.3)
Outpatient	1 (2.2)
Indication/type of infection, *n* (%)	
Pulmonary	12 (26.1)
Febrile neutropenia	11 (23.9)
Fever of unknown origin	9 (15.6)
Sepsis	6 (13.0)
Endocarditis	3 (6.5)
Bloodstream	3 (6.5)
Bone/joint	1 (2.2)
Intra-abdominal	1 (2.2)
Diagnosis made prior to result, *n* (%)	16 (34.8)
Discharged prior to result, *n* (%)	13 (28.9)
Completed treatment prior to result, *n* (%)	7 (15.2)

**Table 2:** Clinically significant mNGS test organism distribution.

Organism	Immunosuppressed	Non-Immunosuppressed	Total *
Total, *n* (%)	22 (81.5)	5 (18.5)	27 (100.0)
Bacteria			
*Streptococcus mitis*	3	0	3
*Bacteroides fragilis*	2	0	2
*Bacillus cereus*	1	0	1
*Bartonella henselae*	1	0	1
*Clostridium neonatale*	1	0	1
*Enterococcus faecium*	1	0	1
*Fusobacterium nucleatum*	0	1	1
*Haemophilus parainfluenzae*	1	0	1
*Klebsiella aerogenes*	0	1	1
*Moraxella lacunata*	0	1	1
*Pseudomonas aeruginosa*	0	1	1
*Staphylococcus epidermidis*	1	0	1
*Streptococcus intermedius*	0	1	1
Fungi			
*Aspergillus* spp.	4	0	4
*Candida* spp.	3	0	3
Viruses			
Herpes simplex virus type 1	3	0	3
Human adenovirus F	1	0	1

* Totals reflect the number of organisms, not individual patients. Table reflects frequency of organism detection.

**Table 3:** Patients with positive clinical impact.

	Indication	Empiric Antimicrobial Regimen	mNGS Result	New Diagnosis	Modifications to Antimicrobial Regimen
1	Sepsis	Cefepime Amphotericin B	*Bacillus cereus* *Bacillus thuringiensis* *Staphylococcus epidermidis*	N/A	• Amphotericin B discontinued
2	Pulmonary	Meropenem	*Escherichia coli* *Haemophilus parainfluenzae*	Endocarditis	• Meropenem de-escalated to ceftriaxone
3	Pulmonary	Meropenem Amphotericin B	No organisms detected	N/A	• Antimicrobials discontinued
4	Febrile neutropenia	Piperacillin–tazobactam Amphotericin B	*Bacteroides fragilis* *Streptococcus mitis* *Staphylococcus epidermidis* *Staphylococcus haemolyticus* *Streptococcus pseudopneumoniae* *Prevotella melaninogenica*	N/A	• Amphotericin B de-escalated to voriconazole
5	Pulmonary	Cefepime Metronidazole Vancomycin Amphotericin B Acyclovir	*Aspergillus terreus* Herpes simplex virus type 1	Aspergillosis	• Amphotericin B de-escalated to voriconazole • Antibiotics discontinued
6	Pulmonary	Cefepime Amphotericin B	*Micrococcus luteus* *Dermacoccus nishinomiyaensis* *Aspergillus flavus* *Aspergillus oryzae*	Aspergillosis	• N/A
7	Febrile neutropenia	Cefepime Metronidazole Vancomycin Amphotericin B	*Streptococcus mitis* *Staphylococcus epidermidis* *Enterococcus faecium*	Bacteremia	• Amphotericin B de-escalated to micafungin
8	Febrile neutropenia	Meropenem Vancomycin Amphotericin B	No organisms detected	N/A	• Antimicrobials discontinued
9	Febrile neutropenia	Meropenem Vancomycin Sulfamethoxazole–trimethoprim	No organisms detected	N/A	• Sulfamethoxazole–trimethoprim dose reduced from treatment to prophylaxis
10	Bloodstream infection	Cefepime Amphotericin B	*Candida dubliniensis* *Candida tropicalis* *Clostridium neonatale* *Streptococcus mitis*	N/A	• Cefepime escalated to piperacillin–tazobactam • Amphotericin B de-escalated to micafungin
11	Febrile neutropenia	Cefepime Vancomycin Posaconazole	No organisms detected	N/A	• Vancomycin discontinued
12	Febrile neutropenia	Cefepime Amphotericin B Posaconazole	No organisms detected	N/A	• Amphotericin B discontinued
13	Febrile neutropenia	Cefepime Amphotericin B Acyclovir	Herpes simplex virus type 1 *Candida albicans* *Bacteroides fragilis*	Candidiasis	• Amphotericin B de-escalated to micafungin
14	Pulmonary	Cefepime Vancomycin Sulfamethoxazole–trimethoprim Fluconazole	*Aspergillus versicolor*	Aspergillosis	• Vancomycin discontinued • Fluconazole escalated to voriconazole
15	Bloodstream infection	Cefepime Vancomycin Micafungin	*Streptococcus mitis*	N/A	• Cefepime and vancomycin de-escalated to ampicillin • Micafungin discontinued
16	Sepsis	Voriconazole	*Klebsiella aerogenes* *Enterococcus faecalis* Cytomegalovirus	N/A	• Voriconazole discontinued
17	Pulmonary	Ceftriaxone	*Streptococcus intermedius*	N/A	• Ceftriaxone de-escalated to ampicillin
18	Endocarditis	Cefepime Vancomycin Rifampin	*Bartonella henselae*	N/A	• Cefepime and rifampin de-escalated to doxycycline and gentamicin

N/A: Not applicable.

**Table 4:** Clinical impact in immunosuppressed patients.

Clinical Impact	Immunosuppressed (*n* = 27)	Non-Immunosuppressed (*n* = 19)	Total (*n* = 46)
Positive Impact, *n* (%)	15 (55.6)	3 (15.8)	18 (39.1)
• New Diagnosis	6 (22.2)	0	6 (13.0)
• Antimicrobial Added	1 (3.7)	0	1 (2.2)
Antibiotic	1 (3.7)	0	1 (2.2)
• Antimicrobial Modified	13 (48.1)	3 (15.8)	16 (34.8)
Escalated *	2 (7.4)	0	2 (4.3)
Antibiotic	1 (3.7)	0	1 (2.2)
Antifungal	1 (3.7)	0	1 (2.2)
De-escalated *	8 (29.6)	1 (5.3)	9 (19.6)
Antibiotic	3 (11.1)	1 (5.3)	4 (8.7)
Antifungal	5 (18.5)	0	5 (10.9)
Discontinuation *	7 (25.9)	2 (10.5)	9 (19.6)
Antibiotic	4 (14.8)	0	4 (8.7)
Antifungal	3 (11.1)	2 (10.5)	5 (10.9)
Dose Adjustment *	1 (3.7)	0	1 (2.2)
Antibiotic	1 (3.7)	0	1 (2.2)
No Impact, *n* (%)	12 (44.4)	16 (84.2)	27 (58.7)

* Patients may be counted more than once if they experienced multiple types of antimicrobial modifications.

**Table 5:** Clinical impact on the type of infection.

Type of Infection	Positive Impact (*n* = 18)	No Impact (*n* = 28)
Pulmonary, *n* (%)	5 (27.8)	7 (25)
Febrile neutropenia, *n* (%)	7 (38.9)	4 (14.3)
Fever of unknown origin, *n* (%)	0	9 (32.1)
Sepsis, *n* (%)	2 (11.1)	4 (14.3)
Endocarditis, *n* (%)	2 (11.1)	1 (3.6)
Bloodstream, *n* (%)	2 (11.1)	1 (3.6)
Bone/joint, *n* (%)	0	1 (3.6)
Intra-abdominal, *n* (%)	0	1 (3.6)

## Data Availability

The data from this study is available from the corresponding author upon reasonable request.

## Abbreviations

CMT	conventional microbiological tests
EHR	electronic health record
ICU	intensive care unit
mNGS	metagenomic next-generation sequencing
PCR	polymerase chain reactions
